# Walking handball as an exercise alternative to conventional walking and recreational team handball

**DOI:** 10.3389/fspor.2026.1784474

**Published:** 2026-03-18

**Authors:** Ricardo Martins, Peter Krustrup, Carlo Castagna, Magni Mohr, Jorge Teixeira, Ivone Carneiro, Susana Póvoas

**Affiliations:** 1 University of Maia, Maia, Portugal; 2 Department of Sports Science and Clinical Biomechanics, SDU Sport and Health Sciences Cluster (SHSC), University of Southern Denmark, Odense, Denmark; 3 Danish Institute for Advanced Study (DIAS), University of Southern Denmark, Odense, Denmark; 4 Sport and Health Sciences, University of Exeter, Exeter, United Kingdom; 5 Department of Education and Sport Sciences, Pegaso Telematic University, Naples, Italy; 6 Centre of Health Science, Faculty of Health, University of the Faroe Islands, Tórshavn, Faroe Islands; 7 Research Center in Sports Sciences, Health Sciences and Human Development (CIDESD), University of Maia, Maia, Portugal

**Keywords:** cardiovascular health, musculoskeletal health, physical inactivity, recreational team sports, walking team sports

## Abstract

**Introduction:**

Recreational team handball (RTH) improves overall health in inactive men and women, yet certain frail populations may be unable to cope with its demands. For them, walking handball (WH) may be a more appropriate alternative. Thus, this study examined the physical and physiological demands and perceived experience of WH, compared with conventional walking modalities [self-paced brisk walking (SPBW) and brisk walking (BW)] and RTH, in middle-aged-to-older men with previous RTH experience.

**Methods:**

A randomized crossover design was applied where twenty-two participants (71 ± 4 years) performed 8 sessions comprising a 10 min warm-up followed by 5 min of rest and 3 × 15 min periods of either SPBW, BW, WH or RTH (2 sessions each), interspersed with 2–3 min breaks. WH and RTH consisted of 5v5 and 6v6 matches on a 40 × 20 m court. Heart rate (HR), blood lactate, activity profile, rating of perceived exertion (RPE), and fun scores were assessed.

**Results:**

WH mean relative HR (67 ± 7%HR_max_) was similar (*p* > 0.05) to SPBW (65 ± 7% HR_max_) and BW (69 ± 7%HR_max_), with RTH (78 ± 9%HR_max_) showing higher values (*p* < 0.05) than all walking modalities, as well as more time spent >90% HR_max_ (RTH: 12 ± 18%; SPBW, BW, WH: 0 ± 0%–2%, *p* < 0.05). Likewise, RTH blood lactate was higher (3.5 ± 1.6 mmol·L^−1^, *p* < 0.05) than all walking modalities, with no differences between them (2.0–2.2 mmol·L^−1^, *p* > 0.05). Distance covered was higher (*p* < 0.05) in SPBW (4,543 ± 429 m) and BW (4,656 ± 383 m) vs. WH (2,561 ± 405 m), although WH elicited a higher number (*p* < 0.05) of accelerations and decelerations across most thresholds. Conversely, fun scores were higher (*p* < 0.05) in WH (7.5 ± 2.2 AU) and RTH (8.3 ± 1.6 AU) vs. SPBW (6.1 ± 2.4 AU) and BW (5.8 ± 2.3 AU), while all RPE scores were lower (*p* < 0.05) in WH vs. SPBW, BW and RTH.

**Conclusions:**

WH elicits moderate-intensity internal load, comparable to conventional walking modalities, with lower total distance covered, but higher frequency and magnitude of accelerations and decelerations, while showing lower RPE, and higher fun scores. This supports its potential to enhance cardiovascular and musculoskeletal health, while fostering long-term exercise adherence. RTH provides the strongest overall training stimulus for fitness and health improvements, with high fun scores, whereas WH may be an alternative or entry pathway for individuals who cannot cope with RTH demands.

**Clinical Trial Registration**: https://clinicaltrials.gov/study/NCT07011290, identifier NCT07011290.

## 1 Introduction

The global population is ageing at an increasing rate. According to the United Nations projections, the number of older adults (i.e., aged 65 years or older) is expected to more than double between 2021 and 2050, rising from 761 million to 1.6 billion (1). This demographic trend has important public health implications, as ageing is associated with a physical, physiological and cognitive decline (2–4). Age-related declines can be attenuated through regular physical activity (PA) (5). In this regard, the World Health Organization (WHO) recommends that adults engage in at least 150–300 min of moderate-intensity aerobic physical activity, or 75–150 min of vigorous-intensity aerobic physical activity per week or an equivalent combination of both, and muscle-strengthening activities to achieve health benefits (6). However, physical inactivity remains a major concern, with an estimated 1.8 billion adults globally failing to meet the PA recommendations (7). Furthermore, in conventional exercise interventions (e.g., aerobic, strength, balance and flexibility training), training sessions attendance can be as low as 58%, with dropout rates reaching up to 35% (8).

Conversely, recreational team sports generally foster higher intrinsic motivation compared to other individual forms of exercise like cycling (9) or resistance training (10), consistently showing high adherence and low dropout rates (11). Moreover, recreational team sports have long demonstrated to provide a large spectrum of health benefits (11, 12), making them highly relevant for the aging population (13). The high intense intermittent nature of recreational team sports, namely recreational team handball (RTH), elicits a notable cardiovascular stress (14, 15), that has been associated with positive cardiorespiratory adaptations (16), which are linked to a reduction in the risk of all-cause mortality (17, 18). Nonetheless, those with physical or health limitations may not be able to cope with these demands. For those individuals who are not attracted to commonly prescribed conventional exercise modalities (e.g., supervised walking or resistance training) but are motivated to engage in recreational team sports, walking sports may represent a suitable exercise alternative.

Recent scoping reviews on walking football have described it as a less demanding exercise format than running football (19, 20). The available evidence, while limited in scale and scope, suggests that walking football elicits cardiovascular loads of ∼80% of maximal heart rate (HR_max_) while maintaining high enjoyment levels and adherence rates (19, 20). However, the current literature on walking sports beyond walking football is non-existent. Consequently, the physical and physiological demands of alternative walking sports, such as walking handball (WH) remain unknown. This lack of empirical evidence limits practitioners' confidence in prescribing WH as a health-promoting walking sport modality similar to walking football (19, 20).

To address this gap, we aimed to characterize WH's internal and external loads and participants' perceived experience. We also sought to compare these demands with conventional walking modalities [i.e., self-paced brisk walking (SPBW) and externally paced brisk walking (BW)] and RTH. It was hypothesized that SPBW would elicit the lowest intensity and WH would present similar intensity to BW, while RTH would remain the most demanding exercise modality.

## 2 Methods

### 2.1 Participants

Twenty-two middle-aged-to-older men (Table 1) were invited and agreed to participate in this study. The inclusion criteria were: 50–80-year-old men with previous experience in recreational team handball (at least 6 months). Participants were excluded if they had any sort of limitation that prevented them from running or gripping the ball used in the recreational handball training sessions, or had medical contraindications to partake in moderate-to-vigorous PA. A detailed description of the study aims, procedures, potential health risks, and benefits was provided to all participants before they signed a written informed consent form, in accordance with the Declaration of Helsinki. Ethical approval was granted by the Ethics and Deontology Council of University of Maia (236/2024). The study was prospectively registered at ClinicalTrials.gov (NCT07011290).

**Table 1:** Participants' characteristics.

Variable	Men (*n* = 22)
Age (years)	71 ± 4 (64–76)
Stature (cm)	171 ± 7 (160–191)
Body mass (kg)	75.3 ± 9.4 (62.3–97.4)
BMI (kg⋅m^−2^)	25.7 ± 2.7 (20.1–31.6)
Fat mass (%)	28.3 ± 4.0 (20.6–36.0)
RHR (b·min^−1^)	66 ± 7 (55–80)
SBP (mmHg)	134 ± 18 (113–189)
DBP (mmHg)	77 ± 8 (63–95)
YYIE1 distance (m)	571 ± 276 (200–1,600)
STS (reps)	28 ± 5 (17–37)
RTH experience (years)	5 ± 2 (3–6)
IPAQ-SF score	
Low (*n*)	3
Moderate (*n*)	8
High (*n*)	11

Values are presented as means ± standard deviation and range. BMI, body mass index; RHR, resting heart rate; SBP, systolic blood pressure; DBP, diastolic blood pressure; YYIE1, Yo-Yo intermittent endurance level 1 test; STS, sit to stand test; RTH, recreational team handball; IPAQ-SF, international physical activity questionnaire-short form.

### 2.2 Study design

**Figure 1:**
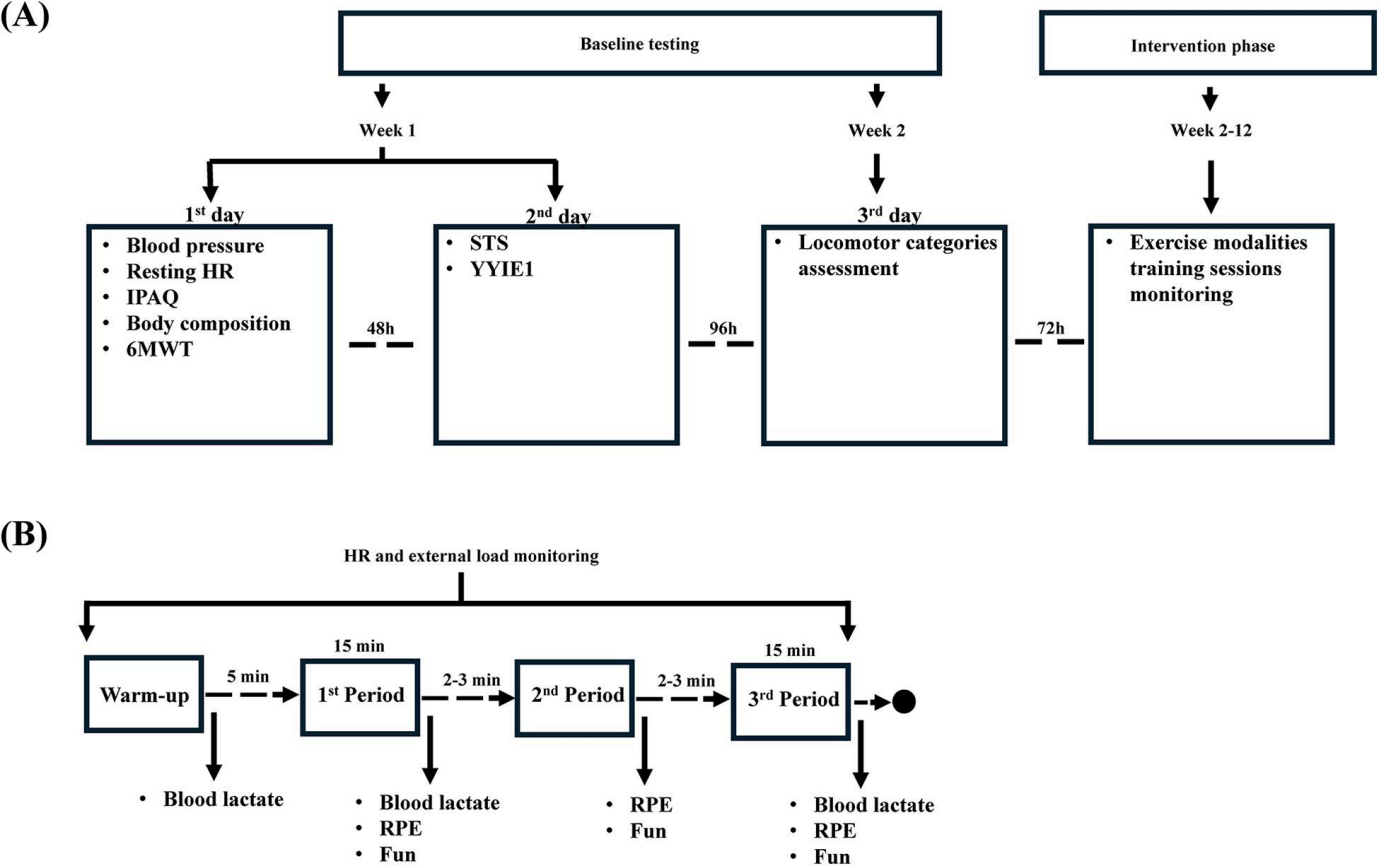
**(A)** study schematic protocol and **(B)** exercise modalities testing and monitoring throughout each training session. IPAQ, international physical activity questionnaire; HR, heart rate; RPE, rating of perceived exertion; STS, sit-to-stand test; YYIE1, Yo-Yo intermittent endurance level 1 test; 6MWT, 6-minute walk test..

A crossover randomized design was implemented in this study. To examine the physical and physiological demands, and perceived experience of each exercise modality, i.e., SPBW, BW, WH and RTH, the participants were randomly assigned to one of two groups. Each group underwent 8 training sessions, 2 of each exercise modality, in a random order. Each training session comprised a single exercise modality (either SPBW, BW, WH or RTH). All sessions began with a standardized 10-min warm-up, followed by 5 min of rest and 3 × 15 min periods of the assigned exercise modality, interspersed with 2–3 min breaks (Figure 1). Training sessions were performed in the morning, with at least 48 h of recovery and at most 7 days apart from the previous training session. Water was provided *ad libitum* before, during, and after the training sessions, to ensure that the participants were properly hydrated.

SPBW and BW involved exclusively continuous walking performed around the indoor team handball court (40 × 20 m), differing only in pace regulation (self-paced following an instruction vs. externally imposed). In contrast, WH and RTH are intermittent exercise modalities played under similar modified team handball rules, with the only difference between the two being that running was prohibited in WH.

The standardized warm-up consisted of runs with a progressive increase in intensity, combined with dynamic stretching of the upper and lower limbs. Balance and strength exercises, such as squats, frontal and side lunges and push-ups, that targeted the main muscle groups activated throughout the sessions, were also performed. The mean heart rate (HR) achieved throughout the warm-up during the exercise sessions was 112 ± 14 b·min^−1^, corresponding to 71%HR_max_.

Conventional walking modalities consisted of SPBW and BW, aiming at characterizing walking at moderate intensity when self-regulated (SPBW) and externally controlled (BW) to achieve the defined intensity. Thus, SPBW speed was self-controlled by each participant, following only the instruction of walking at a pace where they could talk, but not sing, in order to reach moderate intensity levels (21).

Individualized BW speeds were determined during baseline testing and during two additional testing sessions, used to fine-tune the walking speeds necessary to reach, at least, moderate intensity (64%–76%HR_max_) that corresponded to brisk walking (22). These assessments were performed in the two weeks prior to the first exercise modality testing session. Thus, starting from the participants' individual average walking speed achieved in the 6-minute walk test (6MWT) during baseline testing, walking speed was established as 90% of average individual speed in that test. Afterwards, the participants performed 2 testing sessions in which the speed was adjusted, if necessary, to 95% of their average speed in the 6MWT so that, at least, moderate intensity (64%–76%HR_max_) (22) would be achieved during the BW exercise modality testing. Based on the participants' results, two groups were then defined, one group walked at 6.5 km·h^−1^ while the other group walked at 5.6 km·h^−1^ during the BW training sessions, establishing two different walking speeds that came close to 95% of the mean speed reached in the 6MWT by each individual. To ensure that each participant maintained the established speed, a physical education graduate imposed the target walking speed throughout the BW sessions by dictating the participants' walking pace.

In the WH and RTH sessions, specific changes were applied to the official team handball rules (14, 15, 23). The outfield players and the goalkeeper rotated positions every 2 min and no substitutions were allowed. The balls used were softer, lighter and smaller than official team handball balls (47 cm circumference, GOALCHA, Fredericia, Denmark). In addition to the use of specific balls, no physical contact was allowed to reduce the potential risk of contact injuries, while dribbling was also not permitted to increase players' involvement in the match. WH and RTH were played as 5v5 or 6v6 game formats, on a 40 × 20 m indoor court, representing 80 m^2^ or 67 m^2^ per player, respectively. In WH, the participants were not allowed to run, meaning that at least one foot needed to always remain in contact with the ground. SPBW and BW sessions consisted of walking around the indoor court.

### 2.3 Baseline testing

Before the beginning of the baseline testing, the participants were informed and familiarized with the procedures for each test by performing submaximal versions of tests. Furthermore, the participants were instructed not to partake in intense PA in the 48 h prior to the testing days.

On the first day of testing, the participants were asked to fill out a form that included the International Physical Activity Questionnaire-Short Form (IPAQ-SF) (24) to determine PA levels (low, moderate, or high) according to established scoring protocols (25) and a question regarding how many years/months of experience they had with RTH. After, on the same day, anthropometric and body composition assessments were performed including, body mass, stature, body mass index (BMI), fat mass, systolic blood pressure (SBP), diastolic blood pressure (DBP), and resting HR. Following these measurements, the participants performed the 6MWT (26).

Body mass (0.01 kg) and fat mass (%) were determined using a bioimpedance digital scale (Tanita Inner Scan BC 532, Tokyo, Japan), while stature (0.1 cm) was measured using a stadiometer (Seca 213, Hamburg, Germany), based on standardized protocols (27). BMI (kg·m^−2^) was calculated from body mass and stature measurements.

SBP, DBP, and resting HR were measured using an automatic blood pressure monitor (multiparameter patient monitor, Omron Z207, Kyoto, Japan). After ensuring no disturbances could affect the measurements, the participants rested in a seated position for 5 min (28). Subsequently, three measurements were taken with a 1 min interval between them, in a seated and relaxed position. The mean value of the last two measurements was considered (28). For resting HR, the lowest HR value among the three measurements was recorded.

During the 6MWT (26), average walking speed and HR were assessed to determine walking speed during the BW sessions.

On the second day of baseline testing, the participants completed the sit to stand test to assess the neuromuscular function of the lower limbs (29). Thereafter, the Yo-Yo intermittent endurance level 1 test (YYIE1) was performed to evaluate aerobic intermittent exercise performance (30).

### 2.4 Exercise modalities testing

#### 2.4.1 Heart rate measurements

During all training sessions of each selected exercise modality, HR was monitored using HR monitors (Firstbeat Technologies Ltd., version 4.5.0.2, Jyväskylä, Finland). Values are presented as absolute and relative to the HR_max_, determined as the highest value of HR during the YYIE1 or the training sessions, according to a multiple testing approach (31).

#### 2.4.2 Activity profile

Locomotor activities in the four exercise modalities (SPBW, BW, WH and RTH), such as distance, speed, accelerations and decelerations were tracked using the Team Polar Pro system in indoor mode (POLAR, Polar Electro Oy, Kempele, Finland).

Speed thresholds were determined individually for each participant for 5 locomotor zones (walking, fast walking, jogging, fast running and sprinting) using photoelectric cells (Witty System, Microgate, Bolzano, Italy) (Table 2). Participants were asked to perform each locomotor category twice over a 20 m linear course with recovery intervals of ∼90 s to determine individual speed for every locomotor zone (15). Markers were placed 1 m before the starting line and 1 m after the finishing line, with participants instructed to decelerate only after passing that line.

**Table 2:** Mean individual speeds for each locomotor zones.

Locomotor zones	Mean ± SD
Walking	5.6 ± 0.4 km·h^−1^
Fast walking	7.3 ± 0.8 km·h^−1^
Jogging	9.5 ± 0.8 km·h^−1^
Fast running	12.2 ± 1.6 km·h^−1^
Sprinting	16.3 ± 2.6 km·h^−1^

The number of accelerations and decelerations were determined using the following thresholds: 0.5–0.99 m·s^−2^, 1.00–1.99 m·s^−2^, >2.00 m·s^−2^ (32).

#### 2.4.3 Blood lactate

Blood lactate measurements were performed at approximately 3–5 min at the end of the YYIE1, to determine peak blood lactate concentration (BL_peak_) after progressive intensity intermittent exercise performed until exhaustion. This period duration was based on a previous study that assessed blood lactate concentrations in untrained and trained men throughout the Yo-Yo intermittent endurance level 2 test and during recovery (1′, 3′, 5′, 10′ and 15′), finding peak blood lactate values to be reached after 3–5 min of recovery from the test (33).

In each training session, blood lactate samples (30 μl) were collected from the right earlobe (34) in resting conditions (at baseline), immediately after the warm-up (to account for the possible influence of the intensity of the warm-up in lactate accumulation mainly after the first 15 min period), and at the end of the first and third 15 min periods to determine period and session blood lactate values. A portable electroenzymatic lactate device analyser (Lactate Pro 2 LT-1730, Arkray, Amsterdam, The Netherlands) was used for analysing each sample. Blood lactate values are presented as absolute and relative to BL_peak_, obtained during the YYIE1 or the training sessions.

#### 2.4.4 Perceived experience

Respiratory and muscle RPE were assessed along with global RPE to estimate the internal load (35). Fun scores were determined during each exercise modality using a visual analogue scale (0–10 AU) (36). RPE and fun scores were assessed at the end of the first, second and third periods of each training session.

### 2.5 Statistical analysis

All statistical analyses were performed in R (4.5.2 version). Power analysis was performed for the within-subject factor modality (4 levels: SPBW, BW, WH and RTH) using a linear mixed-effects model with subject-specific random intercepts (degrees of freedom via Kenward–Roger/Satterthwaite). Under compound symmetry and equal variances, we calibrated a large standardized omnibus effect (Cohen's *f* = 0.40) at *p* = 0.05 (two-sided). Across plausible within-subject correlations (*r* = 0.3–0.7), the minimum sample required to achieve ≥0.80 power was *N* = 20 participants completing all four conditions. With 22 completers in the present study, the achieved power was approximately 0.84. The normality of the distribution of each variable analysed was assessed using the Shapiro–Wilk test. Descriptive statistics were presented as means ± standard deviations (SD). To analyse the differences between exercise modalities and between periods for all variables, a linear mixed model (LMMs) approach was applied (37) using the *lme4* package in R (38). In the model, the exercise modalities were treated as a fixed effect, while an ID given to each participant was used as a random intercept to account for individual variability. Pairwise *post-hoc* comparisons were performed using estimated marginal means (EMMs) with Tukey adjustment. Effect sizes were calculated as Cohen's *d*, with 95% confidence intervals, using the *effsize* package in R and interpreted as trivial (<0.2), small (0.2–0.5), medium (0.5–0.8) and large (>0.8) (39). Statistical significance was set at *p* < 0.05.

## 3 Results

### 3.1 Physical and physiological demands

#### 3.1.1 Internal load and perceived experience

Exercise modalities descriptive statistics regarding the cardiovascular, blood lactate, RPE and fun measurements are presented in Table 3. Absolute and relative mean HRs were lower in SPBW compared to BW [absolute: *p* = 0.042, *d* = 0.57 medium, 95% CI: (0.13, 1.01); relative: *p* = 0.023, *d* = 0.62 medium, 95% CI: (0.18, 1.06)]. Peak HRs (absolute and relative) were also lower in SPBW than in WH [absolute: *p* = 0.004, *d* = −0.74 medium, 95% CI: (−1.19, −0.30); relative: *p* = 0.002, *d* = −0.77 medium, 95% CI: (−1.21, −0.33)]. Both mean and peak HRs in RTH were much higher than in all other exercise modalities (SPBW, BW, WH) (*p* < 0.0001). Percentage of time spent at ≤60%HR_max_ in RTH was significantly lower compared with SPBW [*p* < 0.0001, *d* = −1.03 large, 95% CI: (−1.47, −0.59)] and WH [*p* < 0.0001, *d* = −0.80 medium, 95% CI: (−1.24, −0.36)]. Inversely, RTH percentage of time spent >80%HR_max_ was found to be significantly higher than all other exercise modalities (*p* < 0.0001).

**Table 3:** Exercise modalities internal load and perceived experience.

Variables	Exercise modalities			
	SPBW	BW	WH	RTH
Cardiovascular demands				
Mean HR (b·min^−1^)	103 ± 13	108 ± 10^a^	105 ± 12	124 ± 17^a^^,^^b^^,^^c^
Mean HR (%HR_max_)	65 ± 7	69 ± 7^a^	67 ± 7	78 ± 9^a^^,^^b^^,^^c^
Peak HR (b·min^−1^)	110 ± 13	114 ± 11	117 ± 14^a^	139 ± 19^a^^,^^b^^,^^c^
Peak HR (%HR_max_)	70 ± 7	72 ± 8	74 ± 7^a^	88 ± 9^a^^,^^b^^,^^c^
Time >80%HR_max_ (%)	5 ± 16	14 ± 26	7 ± 16	47 ± 33^a^^,^^b^^,^^c^
Time ≤ 60%HR_max_ (%)	28 ± 35	17 ± 31	23 ± 32	4 ± 10^a^^,^^c^
Time 61%–70%HR_max_ (%)	45 ± 33	46 ± 41	39 ± 27	17 ± 20^a^^,^^b^^,^^c^
Time 71%–80%HR_max_ (%)	21 ± 29	23 ± 31	30 ± 28	32 ± 21
Time 81%–90%HR_max_ (%)	5 ± 15	14 ± 26	6 ± 14	34 ± 24^a^^,^^b^^,^^c^
Time 91%–100%HR_max_ (%)	0 ± 0	0 ± 1	0 ± 2	12 ± 18^a^^,^^b^^,^^c^
Blood lactate				
Mean blood lactate (mmol^.^L^−1^)	2.0 ± 0.7	2.2 ± 0.7	2.1 ± 0.5	3.5 ± 1.6^a^^,^^b^^,^^c^
Peak blood lactate (mmol^.^L^−1^)	2.2 ± 0.8	2.4 ± 0.9	2.4 ± 0.7	3.9 ± 1.8^a^^,^^b^^,^^c^
Mean blood lactate (%BL_max_)	33 ± 14	35 ± 13	34 ± 11	54 ± 21^a^^,^^b^^,^^c^
Peak blood lactate (%BL_peak_)	37 ± 15	39 ± 16	39 ± 15	60 ± 23^a^^,^^b^^,^^c^
Rating of perceived exertion (RPE)				
Respiratory RPE (AU, 0–10)	5.5 ± 1.8	5.6 ± 1.7	4.5 ± 2.0^a^^,^^b^	6.6 ± 1.7^a^^,^^b^^,^^c^
Muscular RPE (AU, 0–10)	5.5 ± 1.9	5.7 ± 1.8	4.2 ± 2.1^a^^,^^b^	6.2 ± 1.8^c^
Global RPE (AU, 0–10)	5.6 ± 1.7	5.6 ± 1.7	4.6 ± 1.9^a^^,^^b^	6.4 ± 1.7^a^^,^^b^^,^^c^
Fun (AU, 0–10)	6.1 ± 2.4	5.8 ± 2.3	7.5 ± 2.2^a^^,^^b^	8.3 ± 1.6^a^^,^^b^

Data are presented as means ± SD; AU, arbitrary units; BL_peak_, peak blood lactate concentration; HR_max_, maximal heart rate; RPE, rating of perceived exertion.

a Significantly different from SPBW (*p* < 0.05)

b Significantly different from BW (*p* < 0.05)

c Significantly different from WH (*p* < 0.05).

RTH presented significantly higher mean and peak blood lactate concentrations than SPBW (mean: +77%; peak: +63%), BW (mean: +59%; peak: +63%) and WH (mean: +67%; peak: +63%) (*p* < 0.0001), along with significantly higher relative mean and peak blood lactate (*p* < 0.0001). No differences were found in lactate response between the walking exercise modalities.

Respiratory and global RPE were lower in SPBW and BW compared with RTH (*p* < 0.05), while no significant differences were found in muscular RPE between these three exercise modalities. Conversely, WH exhibited lower values in all RPE measures compared to SPBW [respiratory: *p* = 0.001, *d* = 0.86 large, 95% CI: (0.41, 1.30); muscular: *p* < 0.0001, *d* = 0.96 large, 95% CI: (0.51, 1.40); global: *p* < 0.0001, *d* = 0.83 large, 95% CI: (0.39, 1.28)], BW [respiratory: *p* = 0.0001, *d* = 0.95 large, 95% CI: (0.51, 1.40); muscular: *p* < 0.0001, *d* = 1.15 large, 95% CI: (0.70, 1.60); global: *p* < 0.0001, *d* = 0.91 large, 95% CI: (0.46, 1.35)] and RTH [respiratory: *p* < 0.0001, *d* = 1.74 large, 95% CI: (1.26, 2.21); muscular: *p* < 0.0001, *d* = 1.48 large, 95% CI: (1.02, 1.94); global: *p* < 0.001, *d* = 1.56 large, 95% CI: (1.09, 2.02)].

#### 3.1.2 External load

Participants covered a greater total distance during SPBW and BW compared to WH [SPBW-WH: *p* < 0.0001, *d* = 6.44 large, 95% CI: (5.59, 7.28); BW-WH: *p* < 0.0001, *d* = 6.80 large, 95% CI: (5.92, 7.68)] and RTH [RTH-SPBW: *p* < 0.0001, *d* = −4.78 large, 95% CI: (−5.48, −4.09); BW-RTH: *p* < 0.0001, *d* = 5.14 large, 95% CI: (4.42, 5.86)] (Table 4).

**Table 4:** Exercise modalities external load.

Variables	Exercise Modalities			
	SPBW	BW	WH	RTH
Distance and locomotor zones				
Total distance (m)	4,543 ± 429	4,656 ± 383	2,561 ± 405^a^^,^^b^	3,085 ± 565^a^^,^^b^^,^^c^
Walking (m)	3,755 ± 1,157	3,743 ± 1,197	2,112 ± 399^a^^,^^b^	1,713 ± 359^a^^,^^b^
Fast walking (m)	778 ± 1,131	906 ± 1,351	284 ± 245^a^^,^^b^	884 ± 364^c^
Jogging (m)	6 ± 17	0 ± 0	22 ± 51	256 ± 253^a^^,^^b^^,^^c^
Fast running (m)	0 ± 0	0 ± 0	0 ± 1	70 ± 144^a^^,^^b^^,^^c^
Sprinting (m)	0 ± 0	0 ± 0	0 ± 0	20 ± 64^a^^,^^b^^,^^c^
Walking (%)	83 ± 25	81 ± 28	83 ± 9	57 ± 14^a^^,^^b^^,^^c^
Fast walking (%)	17 ± 25	18 ± 28	11 ± 9	28 ± 10^a^^,^^c^
Jogging (%)	0 ± 0	0 ± 0	1 ± 3	8 ± 7^a^^,^^b^^,^^c^
Fast running (%)	0 ± 0	0 ± 0	0 ± 0	2 ± 4^a^^,^^b^^,^^c^
Sprinting (%)	0 ± 0	0 ± 0	0 ± 0	1 ± 2abc
Accelerations				
0.50–0.99 m·s^−2^ (count)	28 ± 37	49 ± 72	169 ± 38^ab^	198 ± 31^a^^,^^b^^,^^c^
1.00–1.99 m·s^−2^ (count)	8 ± 9	11 ± 30	169 ± 37^ab^	209 ± 46^a^^,^^b^^,^^c^
>2.00 m·s^−2^ (count)	0 ± 0	0 ± 0	2 ± 4	16 ± 17^a^^,^^b^^,^^c^
Decelerations				
0.50–0.99 m·s^−2^ (count)	23 ± 32	42 ± 64	132 ± 33^a^^,^^b^	181 ± 40^a^^,^^b^^,^^c^
1.00–1.99 m·s^−2^ (count)	6 ± 8	10 ± 30	193 ± 32^a^^,^^b^	214 ± 36^a^^,^^b^^,^^c^
>2.00 m·s^−2^ (count)	0 ± 1	0 ± 0	9 ± 6^a^^,^^b^	32 ± 19^a^^,^^b^^,^^c^

Data are presented as means ± SD.

a Significantly different from SPBW (*p* < 0.05).

b Significantly different from BW (*p* < 0.05).

c Significantly different from WH (*p* < 0.05).

SPBW and BW also showed a greater total distance spent walking compared to WH [SPBW-WH: *p* < 0.0001, *d* = 2.01 large, 95% CI: (1.53, 2.50); BW-WH: *p* < 0.0001, *d* = 1.99 large, 95% CI: (1.52, 2.47)] and RTH [RTH-SPBW: *p* < 0.0001, *d* = −2.49 large, 95% CI: (−3.00, −1.98); BW-RTH: *p* < 0.0001, *d* = 2.47 large, 95% CI: 1.97, 2.97]. Fast walking distance was similar across SPBW, BW and RTH, with all three surpassing WH (*p* < 0.05).

Both walking and fast walking percentages were similar across the walking exercise modalities (SPBW, BW, WH).

**Figure 2:**
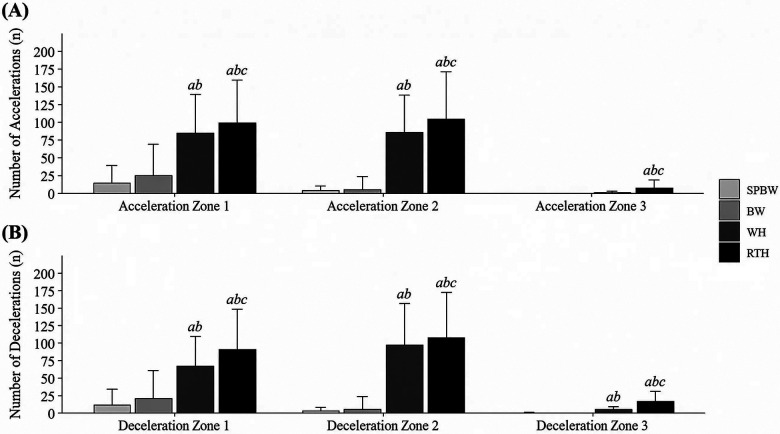
Number of **(A)** accelerations and **(B)** decelerations (count) during the training sessions. Data are presented as means ± SD; Acceleration and deceleration zone 1: 0.50–0.99 m·s^−2^; zone 2: 1.00–1.99 m·s^−2^; zone 3: >2.00 m·s^−2^; a-Significantly different from SPBW (*p* < 0.05); b-Significantly different from BW (*p* < 0.05); c-Significantly different from WH (*p* < 0.05).

**Figure 3:**
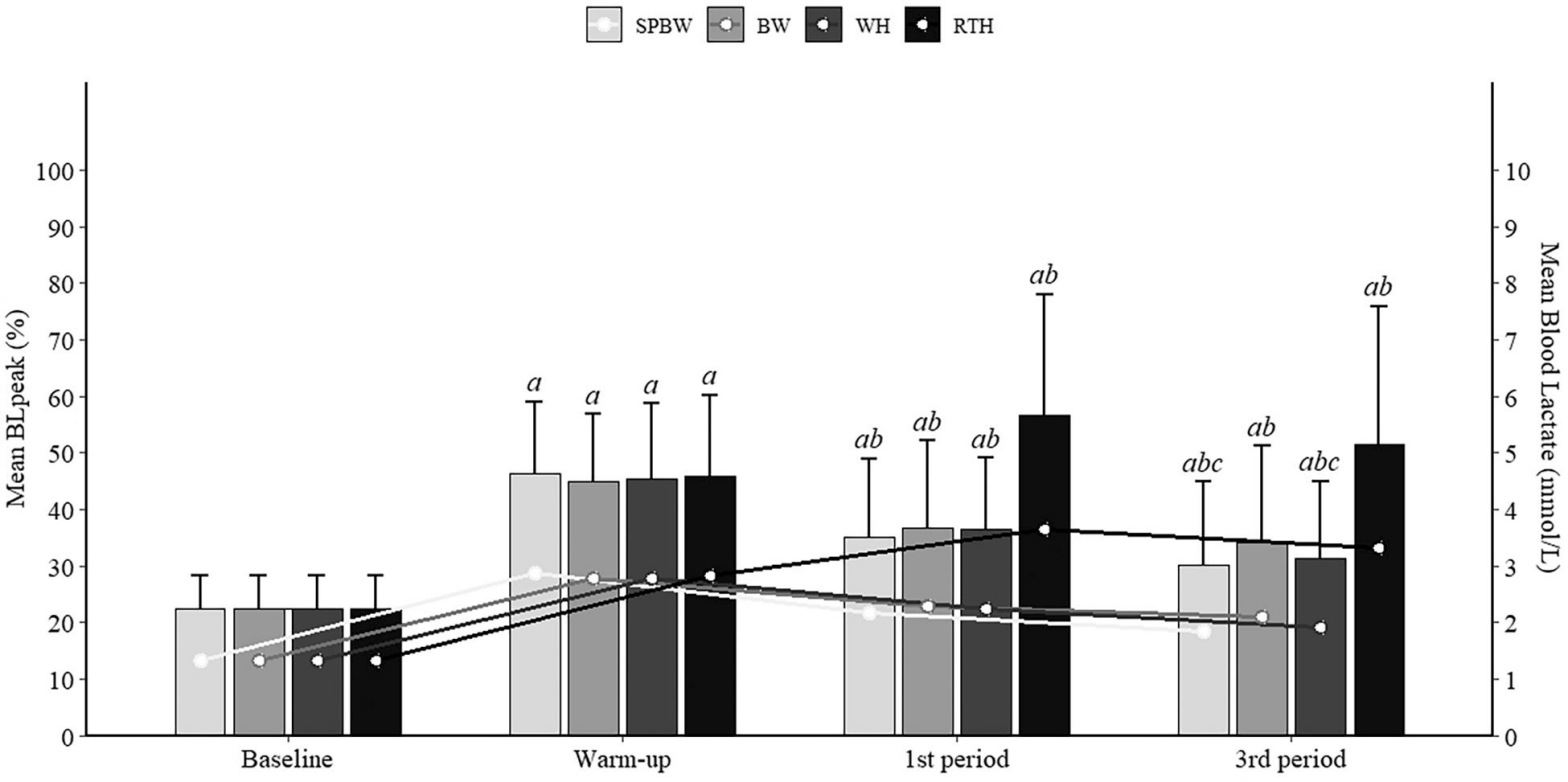
Mean absolute (mmol^.^L^−1^) (lines) and relative (BL_peak_%) (bars) blood lactate across the warm-up, first (1st period) and third periods (3rd period). **a**-Significantly different from baseline (*p* < 0.05); **b**-Significantly different from warm-up (*p* < 0.05); **c**-Significantly different from first period (*p* < 0.05).

For the thresholds of 0.50–0.99 m·s^−2^ and 1.00–1.99 m·s^−2^, WH recorded much more accelerations than SPBW [0.50–0.99 m·s^−2^*: p* < 0.0001, *d* = −3.47 large, 95% CI: (−4.04, −2.89); 1.00–1.99 m·s^−2^*: p* < 0.0001, *d* = −6.29 large, 95% CI: (−7.11, −5.40)] and BW [0.50–0.99 m·s^−2^: *p* < 0.0001, *d* = −2.95 large, 95% CI: (−3.49, −2.41); 1.00–1.99 m·s^−2^: *p* < 0.0001, *d* = −6.18 large, 95% CI: (−6.99, −5.37)] (Figure 2).WH also presented a much higher number of decelerations in all three thresholds (0.50–0.99 m·s^−2^, 1.00–1.99 m·s^−2,^ >2.00 m·s^−2^) compared with SPBW [0.50–0.99 m·s^−2^: *p* < 0.0001, *d* = −2.96 large, 95% CI: (−3.50, −2.42); 1.00–1.99 m·s^−2^*: p* < 0.0001, *d* = −8.48 large, 95% CI: (−9.52, −7.45); >2.00 m·s^−2^*: p* < 0.0001, *d* = −1.03 large, 95% CI: (−1.48, −0.58)] and BW [0.50–0.99 m·s^−2^*: p* < 0.0001, *d* = −2.43 large, 95% CI: (−2.94, −1.93); 1.00–1.99 m·s^−2^*: p* < 0.0001, *d* = −8.27 large, 95% CI: (−9.29, −7.26); >2.00 m·s^−2^*: p* < 0.0001, *d* = −1.04 large, 95% CI: (−1.48, −0.60)] (Figure 3). RTH presented a higher number of accelerations and decelerations than all the other exercise modalities for all thresholds (*p* < 0.05).

### 3.2 Between periods differences

#### 3.2.1 Internal load and perceived experience

During BW, absolute mean HR, increased from the first to the second [*p* = 0.001, *d* = −0.79 medium, 95% CI: (−1.25, 0.34)] and third [*p* < 0.0001, *d* = −1.07 large, 95% CI: (−1.54, −0.61)] periods (Table 5). Relative mean HR remained stable from the first to the third period in SPBW (+1%), BW (+2%) and WH (*p* > 0.05).

**Table 5:** Exercise modalities internal load during each 15 min period.

Variables	Exercise Modalities											
	SPBW			BW			WH			RTH		
	1st	2nd	3rd	1st	2nd	3rd	1st	2nd	3rd	1st	2nd	3rd
Mean HR (b·min^−1^)	102 ± 13	104 ± 13	104 ± 14	106 ± 10	108 ± 11^c^	109 ± 11^c^	105 ± 13	106 ± 13	105 ± 13	123 ± 17	125 ± 19	124 ± 17
Mean HR (%HR_max_)	65 ± 8	66 ± 8	66 ± 8	67 ± 7	69 ± 8	69 ± 8	67 ± 7	67 ± 8	67 ± 7	78 ± 9	79 ± 9	78 ± 9
Peak HR (b·min^−1^)	109 ± 14	110 ± 14	110 ± 15	113 ± 11	114 ± 12	114 ± 12	120 ± 15	118 ± 16	118 ± 16	138 ± 19	140 ± 21	138 ± 20
Peak HR (%HR_max_)	69 ± 8	70 ± 7	70 ± 8	72 ± 5	72 ± 5	73 ± 6	76 ± 8	75 ± 8	75 ± 8	87 ± 9	88 ± 10	88 ± 9
Time >80%HR_max_ (%)	3 ± 16	6 ± 21	6 ± 19	7 ± 19	16 ± 31	17 ± 33	7 ± 15	7 ± 18	6 ± 18	46 ± 33	49 ± 36	45 ± 36
Time ≤60%HR_max_ (%)	33 ± 40	25 ± 36	28 ± 40	26 ± 38	16 ± 33	15 ± 31	23 ± 31	21 ± 35	25 ± 35	8 ± 13	4 ± 16	1 ± 8^c^
Time 61%–70%HR_max_ (%)	47 ± 38	48 ± 41	41 ± 40	41 ± 40	46 ± 44	49 ± 44	39 ± 27	40 ± 31	38 ± 29	42 ± 40	46 ± 43	49 ± 44
Time 71%–80%HR_max_ (%)	17 ± 30	21 ± 34	25 ± 36	25 ± 36	22 ± 34	19 ± 31	31 ± 27	31 ± 30	31 ± 32	30 ± 22	32 ± 22	35 ± 27
Time 81%–90%HR_max_ (%)	3 ± 16	6 ± 21	6 ± 18	7 ± 19	16 ± 31	17 ± 32	6 ± 13	7 ± 15	6 ± 18	35 ± 26	34 ± 28	34 ± 26
Time 91%–100%HR_max_ (%)	0 ± 0	0 ± 0	0 ± 1	0 ± 0	0 ± 0	0 ± 2	0 ± 2	1 ± 4	0 ± 1	11 ± 17	15 ± 22	11 ± 18
Respiratory RPE (AU)	4.4 ± 1.9	5.0 ± 1.8^c^	5.4 ± 1.7^c^	4.8 ± 2.0	5.2 ± 2.0	5.7 ± 1.8^c^^,^^d^	3.8 ± 1.5	4.3 ± 1.3	4.6 ± 1.9	5.4 ± 1.9	6.1 ± 1.8^c^	6.6 ± 1.8^c^
Muscular RPE (AU)	4.2 ± 1.8	5.0 ± 1.8^c^	5.5 ± 1.8^c^	4.7 ± 2.1	5.3 ± 2.0^c^	6.0 ± 2.0^c^^,^^d^	3.5 ± 1.8	4.0 ± 1.9	4.3 ± 1.7^c^	5.0 ± 2.1	5.6 ± 2.1^c^	6.1 ± 1.9^c^
Global RPE (AU)	4.2 ± 1.9	5.0 ± 1.6^c^	5.4 ± 1.8^c^	4.8 ± 2.0	5.3 ± 1.8^c^	5.8 ± 1.8^c^^,^^d^	3.7 ± 1.9	4.3 ± 1.7^c^	4.5 ± 1.9^c^	5.3 ± 2.0	5.8 ± 1.8	6.4 ± 1.8^c^^,^^d^
Fun (AU)	6.3 ± 2.4	6.0 ± 2.4	5.6 ± 2.4	5.9 ± 2.3	5.6 ± 2.3	5.2 ± 2.4^c^	6.6 ± 2.3	7.1 ± 2.2	7.6 ± 2.2^c^	7.6 ± 1.8	7.9 ± 1.9	8.1 ± 1.5

Data are presented as means ± SD; AU, arbitrary units; HR_max_, maximal heart rate; RPE, rating of perceived exertion.

c Significantly different from first period (*p* < 0.05).

d Significantly different from second period (*p* < 0.05).

RTH absolute and relative blood lactate means significantly increased from the warm-up to the first period [*p* = 0.002, *d* = 0.74 medium, 95% CI: (0.30, 1.18)], while in the walking modalities (SPBW, BW, WH) a decrease was observed from the warm-up to the second and third periods (*p* < 0.05) (Figure 3).

SPBW [*p* < 0.0001, *d* = −1.01 large, 95% CI: (−1.46, −0.55)], BW [*p* < 0.0001, *d* = −1.01 large, 95% CI: (−1.55, −0.47)] and RTH [*p* < 0.0001, *d* = −1.18 large, 95% CI: (−1.74, −0.62)] respiratory RPE rose from the first to the third period. Muscular RPE also increased, with higher values in the third period compared with the first for SPBW [*p* < 0.0001, *d* = −1.12 large, 95% CI: (−1.58, −0.66)], BW [*p* < 0.0001, *d* = −1.35 large, 95% CI: (−1.83, −0.88)], WH [*p* = 0.001, *d* = −0.82 large, 95% CI: (−1.28, −0.37)] and RTH [*p* < 0.0001, *d* = −1.02 large, 95% CI: (−1.48, −0.56)]. Global RPE followed a similar trend, increasing from the first to the third period across the exercise modalities (*p* < 0.05). In WH, fun scores increased significantly from the first to the third period [*p* = 0.017, *d* = −0.60 medium, 95% CI: (−1.04, −0.16)], while in BW [*p* = 0.006, *d* = 0.67 medium, 95% CI: (0.22, 1.12)] a decline was observed.

#### 3.2.2 External load

No differences were found between periods in most exercise modalities (Table 6), except for RTH, showing higher total distance covered in the first period compared with the second [*p* = 0.014, *d* = 0.62 medium, 95% CI: (0.17, 1.07)] and third periods [*p* < 0.0001, *d* = 1.12 large, 95% CI: (0.65, 1.59)]. In WH, a decrease was observed in distance spent fast walking in the first compared with the third period [*p* = 0.007, *d* = 0.67 medium, 95% CI: (0.22, 1.13)].

**Table 6:** Exercise modalities external load during each 15 min period.

Variables	Exercise Modalities											
	SPBW			BW			WH			RTH		
	1st	2nd	3rd	1st	2nd	3rd	1st	2nd	3rd	1st	2nd	3rd
Distance and locomotor zones												
Total distance (m)	1,513 ± 164	1,533 ± 132	1,497 ± 197	1,557 ± 132	1,558 ± 127	1,541 ± 145	879 ± 176	857 ± 121	844 ± 119	1,091 ± 201	1,024 ± 198^c^	970 ± 212^c^
Walking (m)	1,220 ± 518	1,241 ± 489	1,293 ± 356	1,261 ± 411	1,251 ± 401	1,230 ± 407	691 ± 234	691 ± 401	715 ± 102	572 ± 127	567 ± 122	575 ± 132
Fast walking (m)	288 ± 438	287 ± 472	202 ± 393	293 ± 467	304 ± 449	310 ± 456	127 ± 123	81 ± 84^c^	78 ± 86^c^	343 ± 126	294 ± 131^c^	247 ± 133^c^^,^^d^
Jogging (m)	0 ± 0	0 ± 0	0 ± 0	0 ± 0	0 ± 0	0 ± 0	18 ± 50	3 ± 8^d^	2 ± 5^c^	107 ± 24	89 ± 23	72 ± 24^c^
Fast running (m)	0 ± 0	0 ± 0	0 ± 0	0 ± 0	0 ± 0	0 ± 0	0 ± 1	0 ± 1	0 ± 0	24 ± 52	23 ± 47	24 ± 48
Sprinting (m)	0 ± 0	0 ± 0	0 ± 0	0 ± 0	0 ± 0	0 ± 0	0 ± 0	0 ± 0	0 ± 0	5 ± 17	8 ± 27	7 ± 23
Walking (%)	79 ± 34	81 ± 32	88 ± 24	82 ± 28	81 ± 28	81 ± 28	78 ± 19	85 ± 8^c^	85 ± 9^c^	54 ± 15	57 ± 15^c^	61 ± 14^c^^,^^d^
Fast walking (%)	21 ± 33	19 ± 32	12 ± 24	18 ± 28	18 ± 28	19 ± 28	15 ± 15	9 ± 9	9 ± 9	31 ± 11	28 ± 11^c^	25 ± 12^c^^,^^d^
Jogging (%)	0 ± 1	0 ± 1	0 ± 0	0 ± 0	0 ± 0	0 ± 0	2 ± 7	0 ± 1^c^	0 ± 1^c^	8 ± 8	8 ± 7	6 ± 6
Fast running (%)	0 ± 0	0 ± 0	0 ± 0	0 ± 0	0 ± 0	0 ± 0	0 ± 0	0 ± 0	0 ± 0	2 ± 4	2 ± 4	2 ± 4
Sprinting (%)	0 ± 0	0 ± 0	0 ± 0	0 ± 0	0 ± 0	0 ± 0	0 ± 0	0 ± 0	0 ± 0	0 ± 1	1 ± 2	1 ± 2
Accelerations												
0.50–0.99 m·s^−2^ (count)	8 ± 11	7 ± 10	7 ± 10	14 ± 28	17 ± 27	18 ± 27	56 ± 18	59 ± 13	54 ± 10^d^	70 ± 13	66 ± 13	63 ± 12^c^
1.00–1.99 m·s^−2^ (count)	3 ± 4	2 ± 3	3 ± 4	3 ± 7	4 ± 9	4 ± 16	58 ± 16	58 ± 13	55 ± 13	76 ± 16	68 ± 16^c^	65 ± 19^c^
>2.00 m·s^−2^ (count)	0 ± 0	0 ± 0	0 ± 0	0 ± 0	0 ± 0	0 ± 0	1 ± 1	1 ± 2	1 ± 1	6 ± 6	5 ± 6^c^	5 ± 5^c^
Decelerations												
0.50–0.99 m·s^−2^ (count)	5 ± 7	6 ± 7	11 ± 26	12 ± 24	15 ± 25	15 ± 25	45 ± 15	46 ± 12	43 ± 11	64 ± 16	60 ± 16	57 ± 13^c^
1.00–1.99 m·s^−2^ (count)	2 ± 4	2 ± 2	2 ± 3	3 ± 8	3 ± 8	4 ± 16	65 ± 16	66 ± 10	63 ± 10	76 ± 13	69 ± 12^c^	69 ± 14^c^
>2.00 m·s^−2^ (Count)	0 ± 0	0 ± 0	0 ± 0	0 ± 0	0 ± 0	0 ± 0	3 ± 2	3 ± 3	3 ± 3	11 ± 7	11 ± 6	10 ± 11

Data are presented as means ± SD.

c Significantly different from first period (*p* < 0.05).

d Significantly different from second period (*p* < 0.05).

Decreases were also observed in the number of accelerations for all three acceleration thresholds (0.50–0.99 m·s^−2^, 1.00–1.99 m·s^−2^, >2.00 m·s^−2^) from the first to the third period (*p* < 0.05) for RTH. Moreover, for 0.50–0.99 m·s^−2^ and 1.00–1.99 m·s^−2^ thresholds, the number of decelerations also declined from the first to the third period (*p* < 0.05) in RTH.

### 3.3 Injury and fall incidence

No injuries were observed during any of the exercise modalities. One fall was reported for RTH, while no falls were observed in the walking modalities (SPBW, BW and WH).

## 4 Discussion

The main purpose of this study was to describe the physical and physiological demands, and the perceived experience of WH. Secondly, it aimed to compare these demands with other conventional exercise modalities, namely, SPBW, BW, which are frequently prescribed for the older population, as well as with RTH. The findings of this study showed that the cardiovascular demands are similar between WH and the other walking exercise modalities (SPBW and BW). The anaerobic metabolism was equitably elicited in the three walking modalities. However, WH elicited higher fun scores and was perceived as less demanding than SPBW and BW, suggesting a higher potential for long-term adherence. Despite covering ∼44%–45% less total distance than the other walking modalities (SPBW and BW), WH elicited a much higher frequency and magnitude of accelerations and decelerations, showing markedly greater potential for musculoskeletal adaptations. The physical and physiological demands seem to be maintained throughout the three 15 min periods in all exercise modalities. In the walking modalities, the perceived effort increased as the 45 min training sessions progressed. Yet, while fun scores also increased in WH, a decrease was observed in SPBW and BW, indicating a potential loss of motivation throughout these two exercise modalities training sessions.

Mean HR during WH was just below 70% HR_max_, similar to SPBW and BW, and significantly lower than RTH at nearly 80%HR_max_. Despite similar mean cardiovascular intensities, WH elicited a higher peak HR than SPBW, suggesting that, like RTH, WH intermittent and intense nature also produces acute peaks of cardiovascular stress. All walking modalities fell within the moderate-intensity range (64%–76% HR_max_) according to the American College of Sports Medicine guidelines (22), whereas RTH reached the vigorous intensity threshold (77%–95% HR_max_). Accordingly, WH (7% ± 16%) and the other walking formats (SPBW: 5% ± 16%, BW: 14% ± 26%) accumulated a much lower amount of time above 80%HR_max_, while RTH elicited a much higher amount time in this HR zone (47% ± 33%), consistent with previous RTH studies (15, 16, 40).

The walking exercise modalities elicited moderate-intensity responses, which have previously been associated with improvements in cardiorespiratory fitness (41, 42). One potential mechanism underlying these adaptations is the predominance of fat oxidation during moderate intensity exercise (43, 44), which has been linked to favourable metabolic changes, that may be linked to improvements in VO_2max_ (45). RTH's vigorous profile and time spent >90%HR_max_ are, on the other hand, associated with VO_2max_ improvements (16). Nevertheless, both moderate and vigorous intensity have been linked to beneficial adaptations in immune, cognitive, and epigenetic health (46–48).

Blood lactate results indicate a much lower glycolytic involvement in WH, SPBW, and BW with peak values between 2.2–2.4 mmol·L^−1^ compared to RTH that elicited mean peak values of 3.9 mmol·L^−1^. The use of the anaerobic energy pathway during exercise, such as in high-intensity training, has been previously reported to induce positive peripheral and central adaptations (49). In addition, lactate is not only recognized as a metabolic byproduct, but also as a key signalling molecule in the brain, as it can cross the blood–brain barrier through the monocarboxylate transporter 1 (50) and activate pathways that enhance the expression of brain-derived neurotrophic factor (BDNF) (51, 52). Moreover, elevated muscle lactate may additionally stimulate mitochondrial biogenesis (53), which has been previously shown after recreational team sports training (54). In this context, the elevated lactate concentrations observed during RTH may provide a more favourable environment for positive neuroplastic and muscle mitochondrial adaptations compared with walking modalities. However, it is important to note that blood lactate is a poor predictor of muscle lactate during team sports (55), and that the association between blood lactate acute responses and these adaptations, specifically regarding BDNF, is not strictly linear (56).

With regard to the perceived experience, WH induced lower RPE than SPBW and BW, and higher fun scores, aligning with previous research on recreational team sports (57, 58). Moreover, a decrease in fun scores was observed throughout SPBW and BW training sessions, while the opposite occurred in both in WH and RTH. Enjoyment in WH appears to derive from its game-like structure and social interaction (59), which may offset some fatigue signalling (57). These features suggest that WH could be well-suited for populations requiring higher intrinsic motivation or in rehabilitation contexts.

Total distance covered in WH was substantially lower than in SPBW and BW (∼44% and 45% lower, respectively). However, the percentage of distance covered in the various locomotor thresholds was similar across the walking exercise modalities, which might lead us to believe that the difference observed in absolute values could have been due to the continuous nature of both SPBW and BW compared to WH's intermittent nature, and to the 2 min goalkeeper change rule applied in both handball modalities.

Conversely, the frequency and magnitude of accelerations and decelerations in WH was much higher than in SPBW and BW. These types of movements impose a distinct mechanical strain that stresses the musculoskeletal system (60, 61) and that may lead to positive adaptations (62). Indeed, there is evidence suggesting that high external load, imposed by accelerations, decelerations, jumps and changes of direction in recreational team sports may promote positive musculoskeletal adaptations compared with other forms of exercise (e.g., cycling, resistance exercise, running) (63–65), even among older populations (66).

Horizontal accelerations demand high levels of propulsive horizontal force (67). Rapid concentric actions are associated with a greater ability to rapidly utilise available force and higher early-phase neuromuscular activation (68), thereby increasing power output. These rapid force actions rely increasingly on enhanced neural drive and recruitment/activation of higher-threshold motor units (69). Accordingly, the greater frequency and magnitude of accelerations and the high-force eccentric braking during decelerations in WH and RTH may indicate a greater neuromuscular stimulus compared with SPBW and BW. This potentially involves the recruitment of higher-threshold motor units (fast-twitch fibers), with RTH eliciting the greatest demand out of all modalities.

This is particularly relevant in the context of ageing, as it is associated with a progressive decline in the number of type I and, particularly, type II muscle fibres, largely due to motor unit denervation (70). Interestingly, long-term intense exercise has shown to lead to a reinnervation of muscle fibres, thereby preserving muscle function and structure, while decreasing functional decline (71). WH and RTH combined fun scores and external loads, particularly frequent accelerations and decelerations, may make these modalities especially effective for promoting long-term maintenance of muscle health.

In this regard, although higher distances were covered over the same amount of time in SPBW and BW than in WH, the higher frequency and magnitude of accelerations and decelerations experienced during WH training sessions suggests a greater musculoskeletal loading which may lead to improvements in key bone and muscle health markers as previously addressed. In line with this hypothesis, accelerations and decelerations performed during recreational football were directly associated with changes in lower limb bone mineral density in prostate cancer patients (72). Nevertheless, WH elicited lower overall external loads than RTH, and given the biomechanical differences between walking and running (73), its potential impact on musculoskeletal health remains uncertain. Therefore, future research is needed to clarify whether the external loads imposed by WH, specifically accelerations, decelerations and changes of direction, are sufficient to induce meaningful musculoskeletal adaptations. Thus, despite the lower distance covered during WH in comparison with SPBW and BW, the much higher frequency and magnitude of accelerations, decelerations and possibly multidirectional movements likely increased the mechanical inefficiency of locomotion and the associated metabolic cost. This is consistent with evidence showing elevated energetic demands during non-forward locomotion (74) and the disproportionate metabolic impact of repeated accelerations during intermittent, multidirectional team-sports (75).

Contrary to our hypothesis, the target intensities were similarly achieved during SPBW and BW. This may be due to the fact that these participants have been training regularly RTH for an average of 5 years, which may enable them to more accurately determine the necessary walking speed to reach a moderate intensity effort and also provided them with the discipline and rigor necessary to maintain it during the 3 × 15 min periods, even when their effort was not being externally controlled.

From a practical standpoint, these findings suggest that the internal load elicited in each of the four exercise modalities can promote distinct positive adaptations depending on the main objectives warranted. RTH may be more adequate from a cardiovascular and musculoskeletal perspective compared with the walking exercise modalities, specifically for trained and active individuals, while the walking exercise modalities may display an advantage due to a lower cardiovascular and musculoskeletal strain, possibly reducing adverse events occurrence in more health-sensitive individuals (76). No injuries were observed in any exercise modality, and only one fall occurred during RTH. Although long-term injury risk cannot be ascertained, these results suggest that WH can be safely implemented under supervised conditions without an increased incidence of acute adverse events. In general, these findings are in line with the WHO PA guidelines for adults and older adults (6), which recommend regular engagement in moderate-to-vigorous intensity aerobic activity, complemented by muscle-strengthening and multicomponent exercises. All four exercise modalities in the present study elicited internal loads corresponding to moderate-to-vigorous intensity PA, as reflected by the perceived exertion that was within the recommended range (≥5 RPE) (6). Notably, WH and RTH programmes may additionally fulfil the criteria for multicomponent exercise, as their nature incorporates endurance, resistance and balance training components.

Furthermore, WH may hold a particular value in rehabilitation settings. For example, in patients with chronic conditions such as cancer, where cancer-related fatigue often represents a major barrier for PA (77), the enjoyable and low perceived demanding nature of WH could facilitate sustained participation in exercise programmes. This may help patients to remain active despite treatment side-effects where cancer-related fatigue is present, such as in chemotherapy (78, 79), thereby possibly helping to maintain physical activity levels and counteract some of the negative effects that cytostatic agents impose on the mitochondria (80, 81). Moreover, the described moderate internal load in WH may also be feasible and beneficial for patients with cardiovascular disease, particularly those with low physical fitness levels (82). For these patients, WH could serve as an introductory and transitional exercise modality, allowing for gradual improvements in their physical fitness levels, preparing them to tolerate higher cardiovascular loads as the ones present in RTH. Nonetheless, these patients would need to meet minimal motor requirements, including the ability to walk and receive the adapted team handball ball (softer and lighter) to potentially benefit from a WH-based rehabilitation intervention.

The strengths of this study were its experimental design, namely the standardization of sessions duration (45 min) along with the strict rules for each exercise modality (e.g., in WH one foot needed to always remain in contact with the floor and in SPBW and BW interactions between participants were discouraged) to ensure comparability across the four exercise modalities. Moreover, randomization of both group allocation and the order of exercise modalities performed further reduced potential bias. Furthermore, adequate recovery intervals (minimum 48 h, usually 7 days) between sessions minimized residual fatigue and carry-over effects. The use of a multiple approach (31) to determine the HR_max_ increased the accuracy of the obtained HR values. Importantly, all exercise modalities were performed under standardized warm-up protocols and monitored hydration status, controlling for possible confounding factors.

This study also shows limitations. The external load was assessed using the Team Polar Pro system in the indoor mode using the H10 sensor, which, although used in indoor sport settings, has been reported to exhibit reduced accuracy for distance estimation at higher locomotor speeds (83). However, given the repeated-measures study design, any systematic measurement error is likely to have been consistent across experimental conditions and therefore partially controlled when comparing exercise modalities. Moreover, higher locomotion speed occurred mainly during RTH, suggesting that any potential under or overestimation of distance covered in those zones is unlikely to meaningfully affect the main findings of the study. Furthermore, the previous experience in RTH could have influenced the perceived experience of the participants.

## 5 Conclusions

This study showed that WH elicits moderate cardiovascular load similar to conventional walking, combined with higher frequency and magnitude of accelerations and decelerations, lower perceived effort, and higher enjoyment. These features highlight WH's potential for promoting positive cardiorespiratory and musculoskeletal adaptations and maintaining long-term adherence to exercise.

From a practical perspective, within the exercise modalities analysed in this study, RTH remains the optimal exercise modality to improve fitness levels and overall health profile, while WH could be a safe and engaging alternative for individuals unable to cope with the demands of RTH. WH could also be an introductory option at the beginning of an RTH-based exercise programme, preparing the participants for increasing load as the intervention progresses.

Future research should examine the fitness and health effects of WH-based interventions in untrained and clinical populations, clarify the dose–response relationship (i.e., frequency, duration, and progression) and evaluate this exercise modality feasibility, safety, and adherence, while identifying the key external and internal load determinants underpinning these responses.

## Data Availability

The datasets generated and analysed in this study are not publicly accessible due to confidentiality requirements outlined in the participants’ signed consent forms. Given the small sample size and the detailed nature of the physiological and activity data collected, publicly sharing the dataset could compromise participant anonymity. Although we support principles of research transparency, participant privacy and ethical obligations take priority. Access to the data may be granted upon reasonable request and will require adherence to strict confidentiality procedures. Researchers interested in exploring potential access should contact the corresponding author at spovoas@umaia.pt.
